# Migrated Foreign Body Perforating the Colon: Scope for Colonoscopy

**DOI:** 10.7759/cureus.78112

**Published:** 2025-01-28

**Authors:** Aparimita Das, Tarun S Joseph, Gangireddy Siva Sankar Reddy, Amrit Pipara, Sumit Mukhopadhyay

**Affiliations:** 1 Gastroenterology, Tata Medical Center, Kolkata, IND; 2 Gastroenterology and Hepatology, Tata Medical Center, Kolkata, IND; 3 Surgical Gastroenterology, Tata Medical Center, Kolkata, IND; 4 Radiology, Tata Medical Center, Kolkata, IND

**Keywords:** colon, colonoscopy, hysterectomy, intrauterine device, migration, perforation

## Abstract

Intrauterine devices (IUDs) are still commonly used as a method of contraception in many parts of the world. Bowel perforation is a serious complication of IUD use. In most cases of migration, the IUD is removed laparoscopically or by laparotomy. In this case report, we have documented a novel case of copper IUD (Cu-T) migration and sigmoid colon perforation more than 30 years after insertion with nonspecific symptoms. It was removed colonoscopically without complications or the need for surgical intervention. We recommend the colonoscopy approach as the treatment of choice in similar cases.

## Introduction

Intrauterine devices (IUDs) have been a popular method of contraception and continue to be commonly used. Copper-T (Cu-T) is more popular due to its efficacy, reversibility, lower cost, and fewer complications [1]. Some of the complications of IUD insertion include heavy menstrual bleeding, infection, expulsion, and, in rare instances, perforation and migration. It is therefore important to discuss its complications, including the less common ones. Over time, the number of documented cases of IUD migration has significantly increased, possibly due to a period of increased IUD insertions. An IUD can migrate into the abdomen and perforate adjacent organs causing fistulation, abscess formation, and even obstruction [2-5]. It was observed that Cu-T was more likely to form adhesions upon migration compared to other IUDs, making the conversion of laparoscopic removal to laparotomy more common [6]. A review showed that about 31% of patients with IUD perforations were asymptomatic, resulting in late presentation and a delayed diagnosis [7]. In a retrospective study, the overall incidence was 0.4 to 1.2 per 1000 insertions. Among these, the most common site of perforation was the bowel (32%), followed by the urinary bladder (24%). Other serious complications as a result of perforation include urosepsis, hydronephrosis, and small bowel obstruction, in some cases requiring nephrectomy or bowel resection. While laparoscopic removal of the migrated IUD is still the method of choice, endoscopic removal has been successfully attempted in a few cases [5,7]. This provides a minimally invasive, endoscopic alternative to laparoscopic retrieval and is safer with fewer post-procedure complications. This is a case report outlining a late presentation of Cu-T migration and subsequent bowel perforation that was removed colonoscopically without post-procedure complications.

## Case presentation

The patient was a 64-year-old multipara female with a history of IUD insertion more than 30 years ago. She subsequently underwent a total abdominal hysterectomy in 2007, more than a decade after the insertion of the IUD at which time the IUD was not detected. She had no known comorbidities. She has been asymptomatic for more than 15 years and now presented with on-and-off lower abdominal pain for a year that worsened over the last two weeks. She had a history of fever and left iliac fossa tenderness on examination.

**Figure 1:**
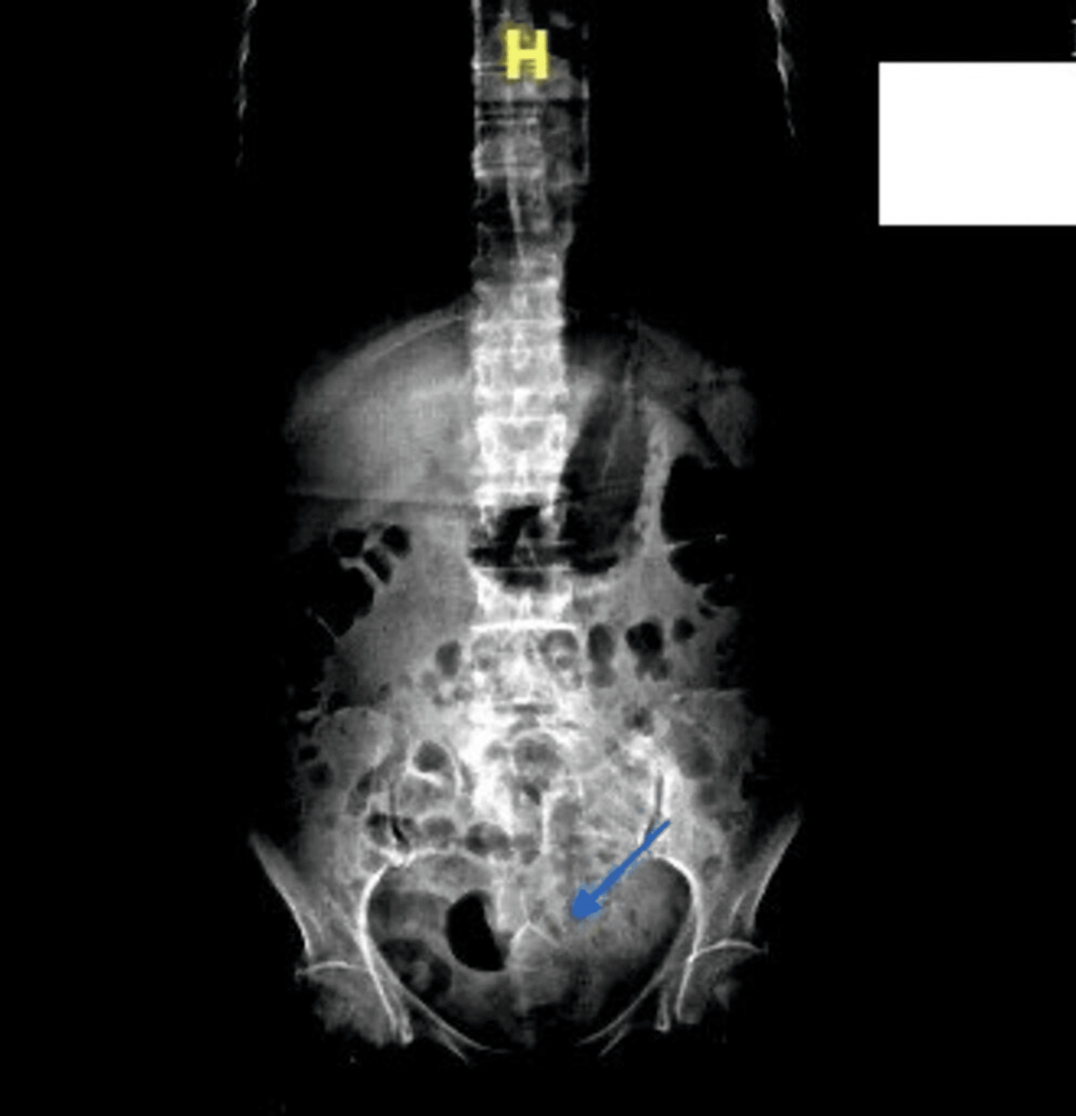
Plain radiograph of the abdomen on presentation showing a T-shaped foreign body.

**Figure 2:**
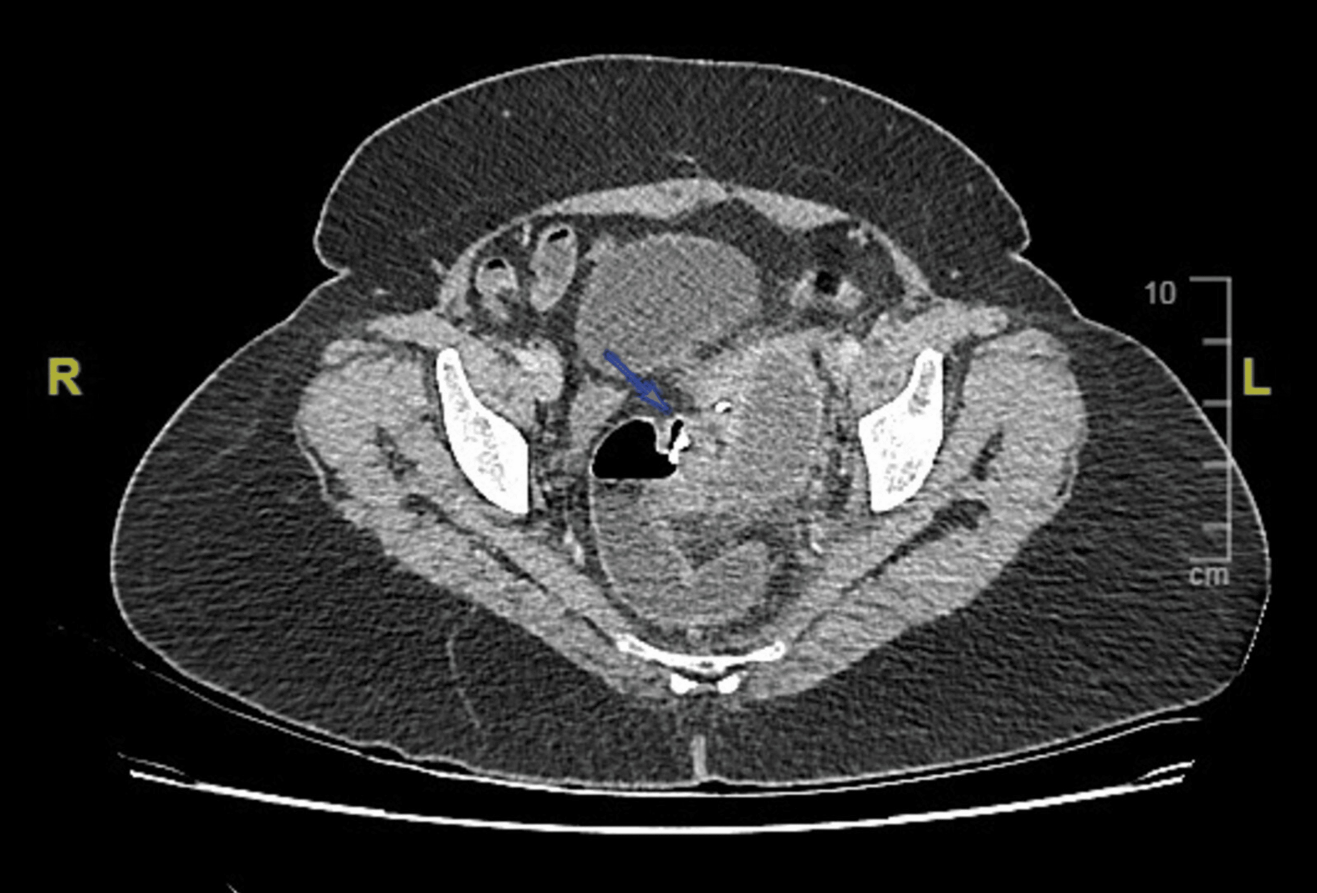
Axial section of CT of the abdomen showing sealed-off sigmoid colon perforation with a foreign body.

**Figure 3:**
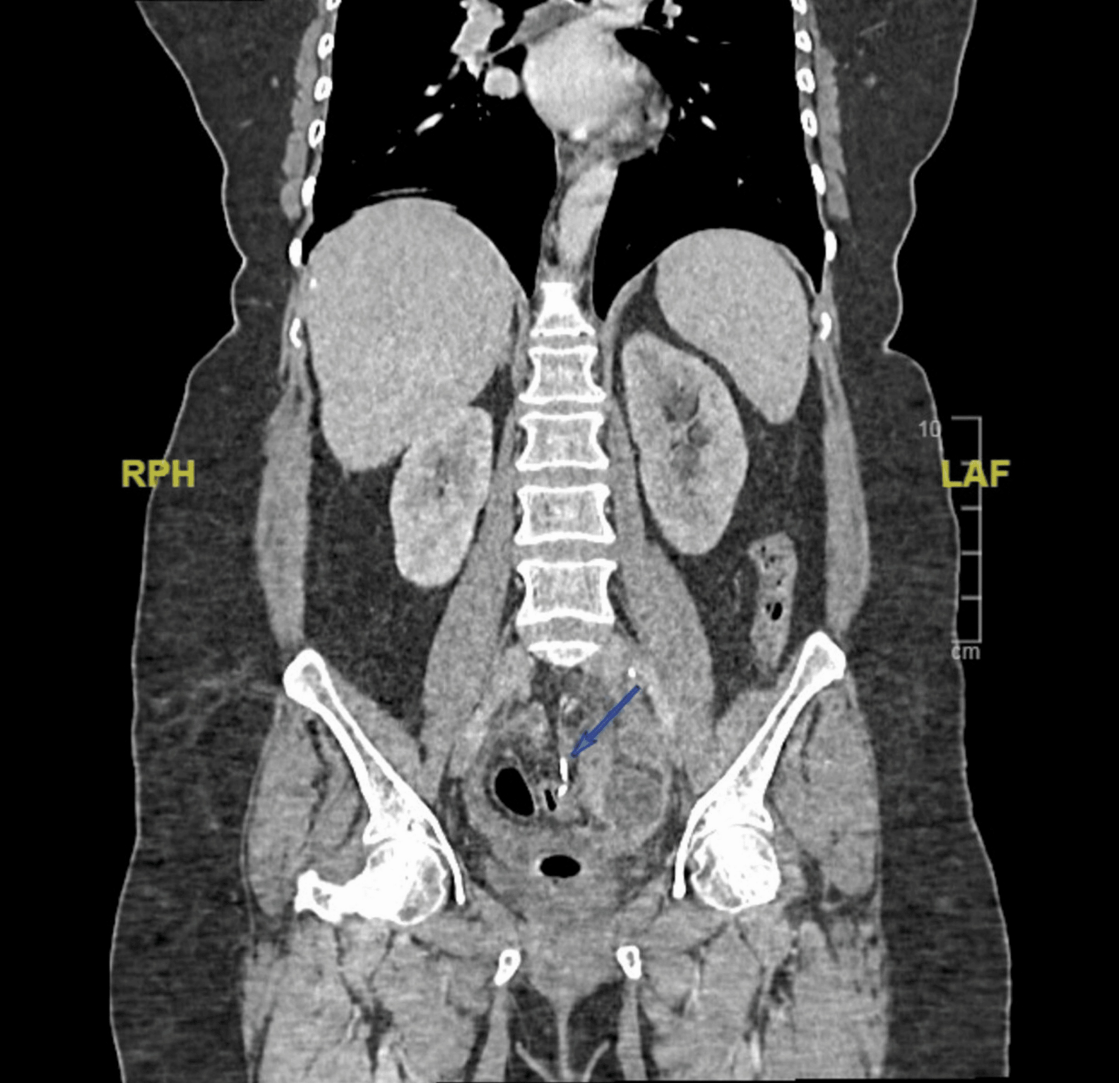
Coronal section of CT imaging showing the hyperdense foreign body.

Initial imaging included an abdominal radiograph and CT imaging, distinctly showing a T-shaped foreign body. It showed a sealed-off sigmoid colon perforation with a hyperdense foreign body and a heterogeneous collection with features of diverticulitis (Figures 1-3).

A recent colonoscopy confirmed a foreign body in the sigmoid colon with surrounding edema. Her IUD had migrated and resulted in a sigmoid colon perforation with one arm embedded in the wall of the colon. The initial suggested approach at other centers was laparoscopic removal with the possibility of a colostomy.

**Figure 4:**
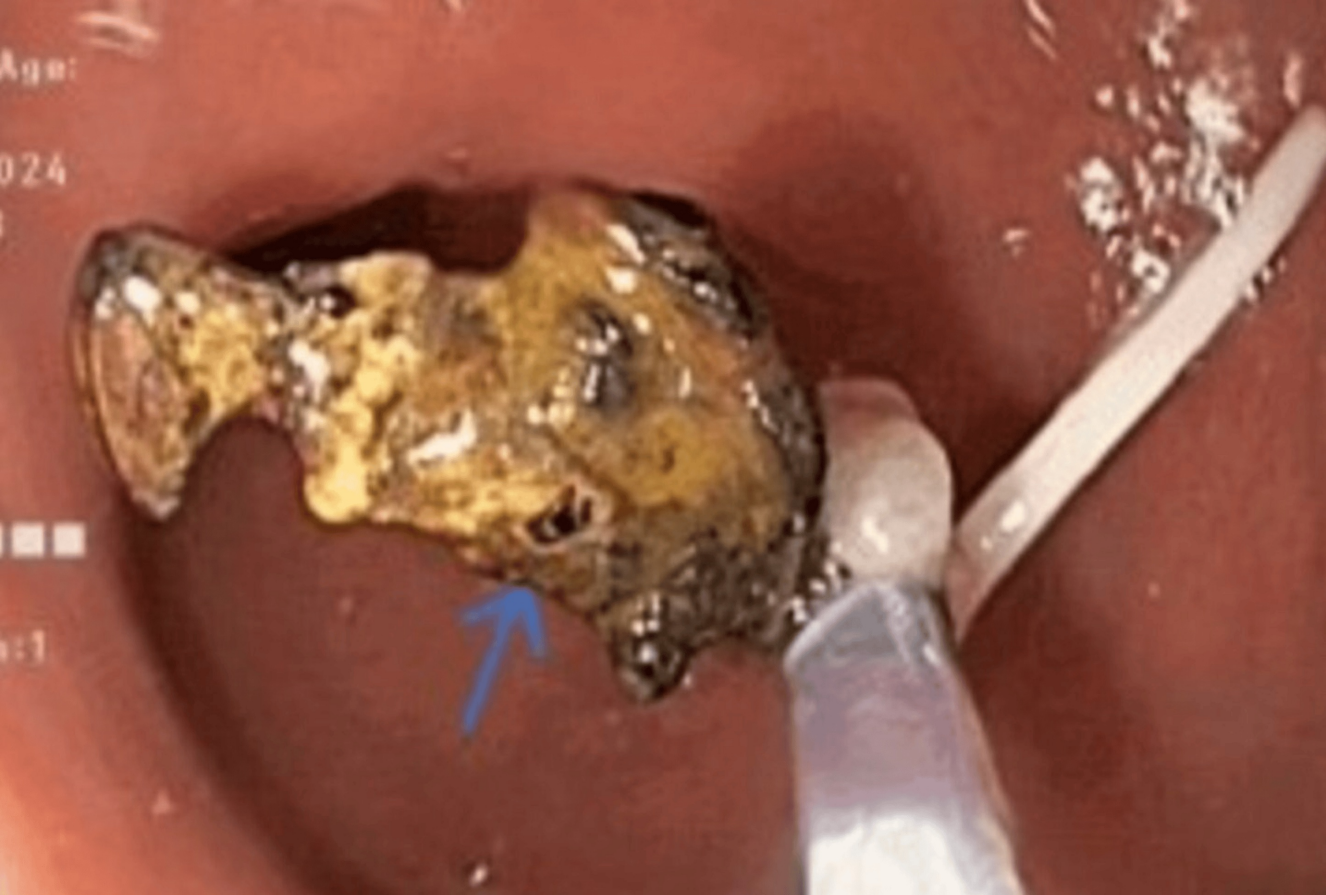
Migrated intrauterine device as seen on colonoscopy.

**Figure 5:**
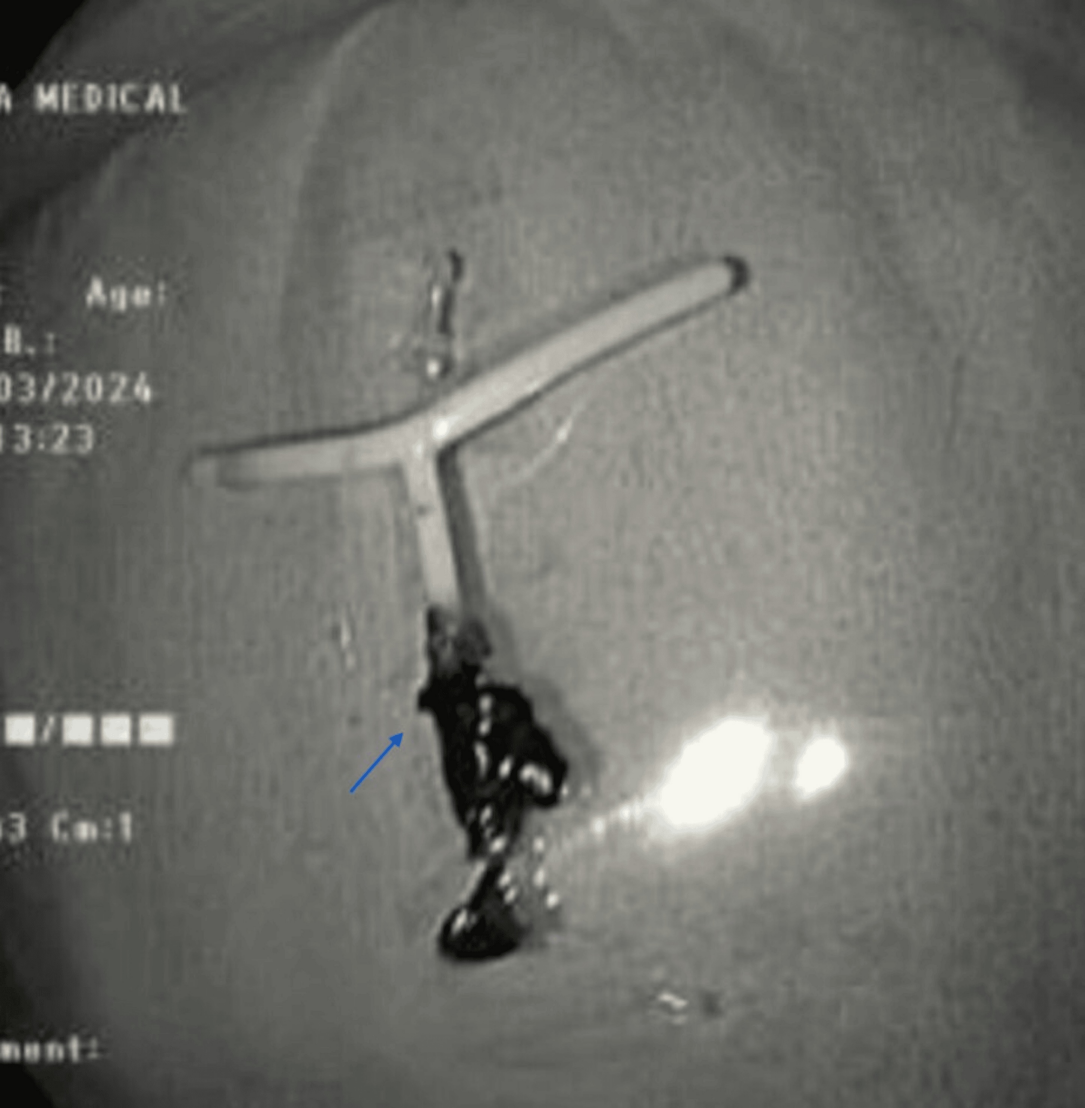
Removed copper T intrauterine device.

**Figure 6:**
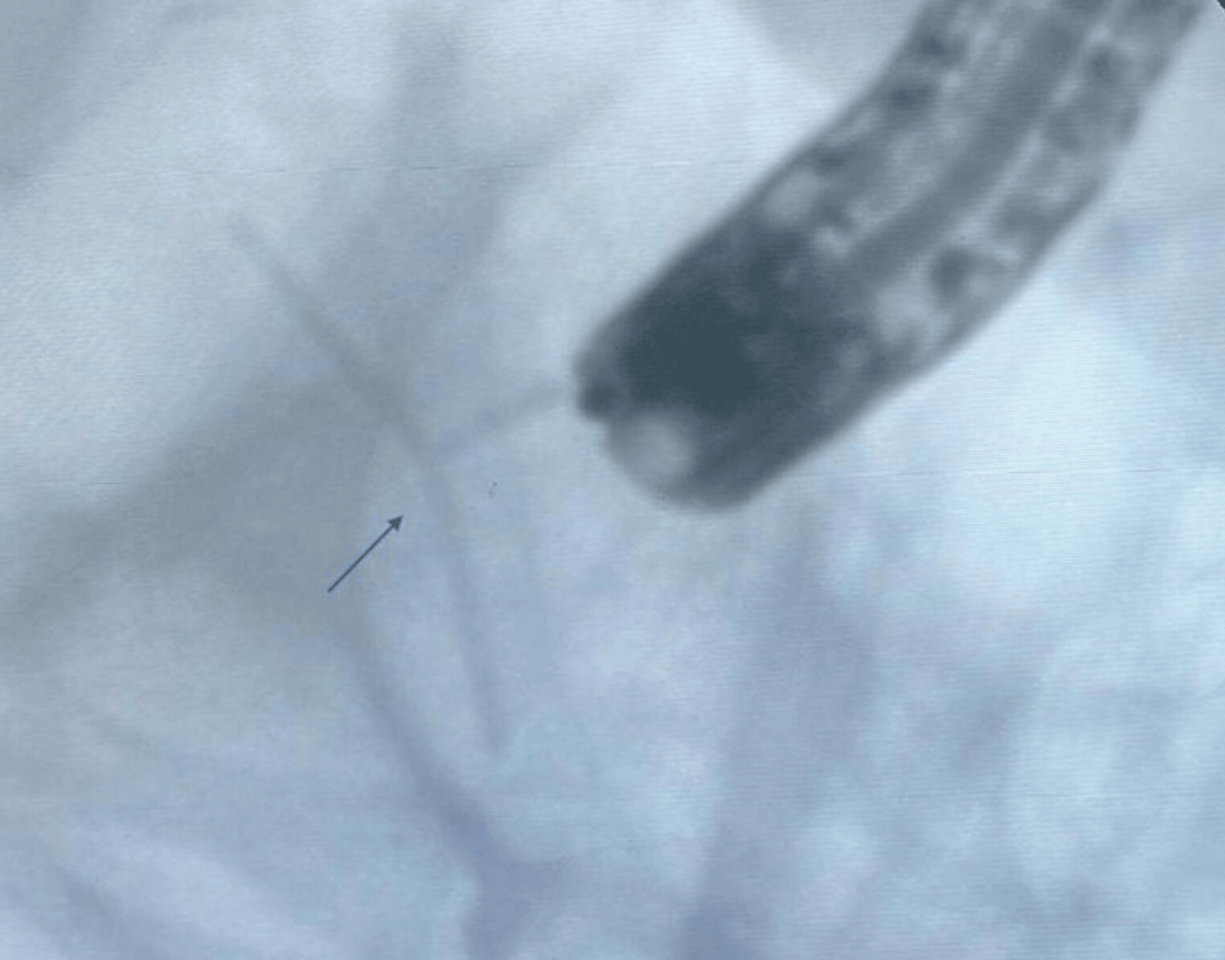
Intrauterine device as seen on fluoroscopy.

After obtaining informed consent, colonoscopic removal of the foreign body was carried out. The foreign body was visualized endoscopically and the blackish foreign body was held using a snare and slowly dislodged from the colonic wall under fluoroscopic guidance wherein all three limbs of the foreign body were removed in its entirety (Figures 4-6). The removed foreign body was the displaced Cu-T that was inserted over three decades prior to the procedure. The procedure was uneventful with no immediate complications. The extended duration post migration and subsequent perforation likely helped to contain the IUD and prevent peritonitis. The resulting defect was approximated using hemoclips. This further helped to prevent complications.

**Figure 7:**
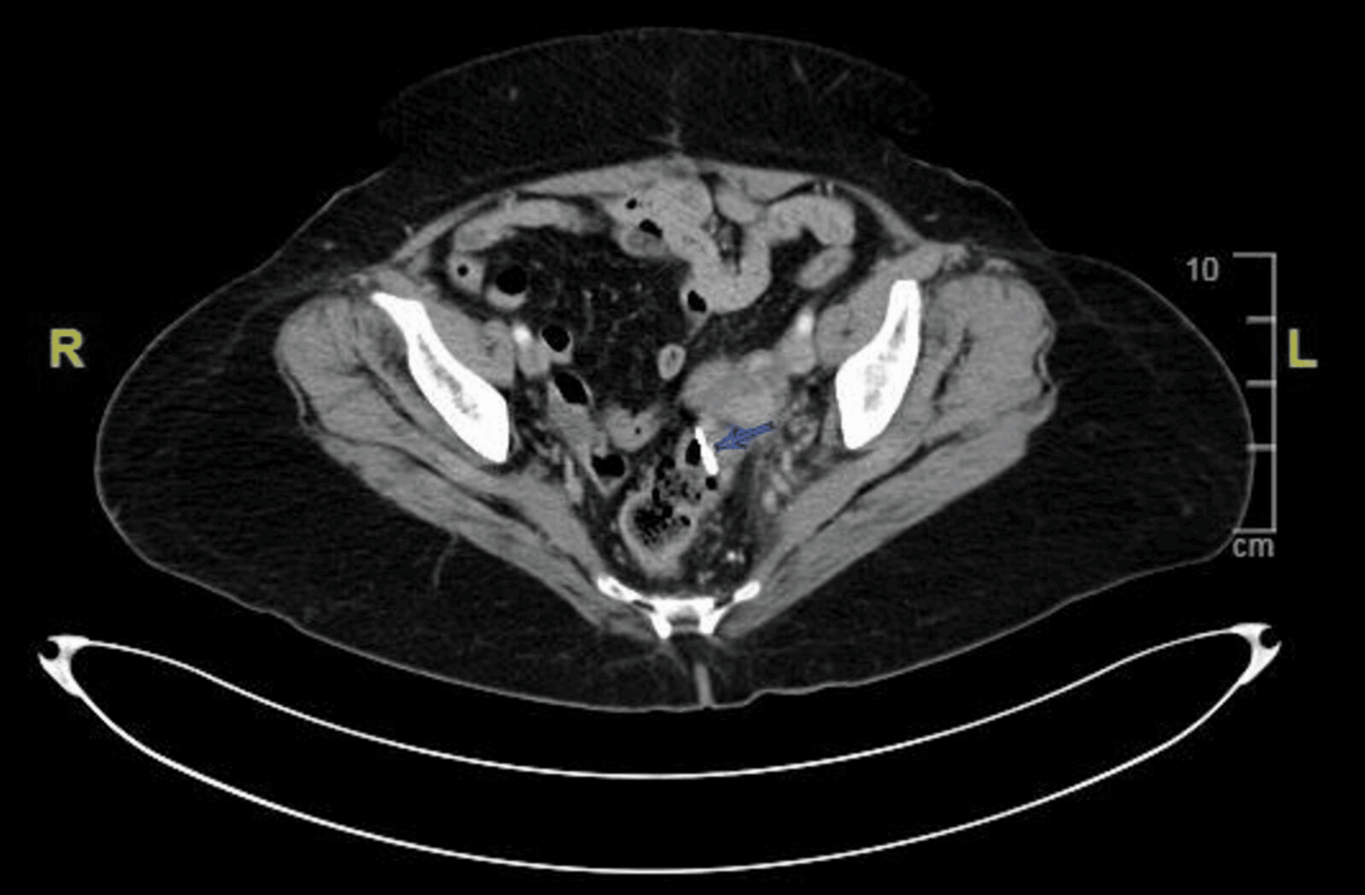
Repeat CT imaging of the abdomen showing hemoclips.

Another CT of the abdomen was done post procedure, which showed the site of defect with the hemoclips (Figure 7).

She was gradually started on a soft diet and discharged in a hemodynamically stable condition, three days following the procedure.

## Discussion

Uterine perforation and subsequent migration is a potentially serious complication of IUD use. Reported incidences range from 0.3 to 2.2 per 1000 insertions for copper IUDs. In a study conducted in a medical college, two out of 240 CuT insertions resulted in migration. IUDs are known to perforate visceral organs, even though the exact mechanism is not clear. Migration has been linked to several factors, including uterine anomalies, prior operations, uterine size, timing of insertion, multiparity, and breastfeeding at the time of insertion. Given the risks posed by migrating IUDs, their removal has been recommended by the World Health Organization. Among the diagnostic modalities, abdominal CT imaging is considered the gold standard; however, transvaginal or abdominal ultrasound involves no radiation exposure and is more cost-effective. Of the methods used to remove IUDs, laparoscopy is the method of choice but it has been found to have a relatively higher rate of conversion to laparotomy, about 68% for IUDs embedded in the bowel, and may even be unsuccessful in some cases where the IUD has migrated into the retroperitoneum. In another study, the conversion rate to laparotomy was 11% where nearly 70% of women underwent laparoscopic removal [6]. Repeat laparoscopy has been required in several instances. Laparotomy, commonly performed due to adhesions, can involve bowel resection and stoma creation, resulting in poor quality of life for the patient. In the European Active Surveillance (EURAS) IUD prospective cohort study, most IUDs were removed laparoscopically. There have been some cases where a combination of endoscopic and surgical techniques was used after a few unsuccessful attempts at retrieval endoscopically [8,9]. However, IUD migration rarely presents as an acute emergency and patients are generally symptomatic after a few months to a few years. This results in chronic inflammation within the peritoneum causing the formation of adhesions and for the IUD to be sequestered. This increases postoperative complications and the need for stoma creation. With advanced endoscopic equipment and expertise, there are several documented cases of successful IUD retrieval, specifically colonoscopy in cases of bowel perforation, thereby avoiding complications of surgery [3,7,10-15].

## Conclusions

Migration of an IUD can lead to grave complications and a careful workup is required on suspicion of migration. However, in cases of IUD migration, each case must be carefully assessed for the feasibility of endoscopic removal. In cases with colon perforation, similar to our case, colonoscopic removal could not only be performed with ease but with no post-procedure complications. Overall the colonoscopy approach is less invasive and has fewer complications than the surgical approach and can be considered as a first-line approach in select cases.
